# Editorial: Valve Virtuosi and Pacer Placers — Reducing the Need for Permanent Pacemaker Implantation After Transcatheter Aortic Valve Replacement

**DOI:** 10.1016/j.shj.2022.100047

**Published:** 2022-06-11

**Authors:** Neal S. Kleiman

**Affiliations:** Department of Cardiology, Section of Interventional Cardiology, Houston Methodist DeBakey Heart and Vascular Center, Houston, Texas, USA

Cardiologists often stumble when deciding whether to implant permanent pacemakers in patients who have undergone transcatheter aortic valve replacement (TAVR). No one particularly wants to implant a permanent pacemaker after TAVR, given the potential detrimental effects on left ventricular function and added resource use as well as observations that AV conduction is frequently found to have recovered in the months after implant.^1^ However, the consequences of overlooking a potentially lethal episode of complete heart block are devastating, particularly after an otherwise successful procedure. While the frequency of permanent pacemaker implantation during hospitalization has declined slightly during the past few years,^2^ it is uncertain whether this phenomenon is truly due to a decrease in the perceived need for a pacemaker or simply a redistribution in the timing of pacemaker implantation resulting from a trend toward earlier hospital discharge.^3^

High-grade atrioventricular block is a well-recognized complication of TAVR (about 9%-15% with commercially available valves) and is a direct consequence of the proximity of the left bundle of His to the basal aortic plane, but there are few rigorously collected data available to guide the decision to implant a pacemaker. While a meta-analysis indicated that permanent pacemaker implantation is associated with increased mortality and rehospitalization for heart failure 1 ​year after TAVR,^4^ a recent report from the Swedish web-based system for enhancement and development of evidence-based care in heart disease evaluated according to recommended therapies registry indicated that adjusted mortality and heart failure hospitalization rates at a mean of 3 ​years were similar for patients with and without pacemakers.^5^ These issues may have been less important when TAVR was restricted to very elderly patients with limited survival, but will become much more pressing as TAVR is extended to patients at low surgical risk (i.e., younger patients) and possibly to those at earlier stages of the aortic stenosis disease process. In 2019, the American College of Cardiology produced an Expert Scientific Panel document,^6^ and in 2020, it produced an Expert Consensus Decision Pathway^7^ intended to guide diagnosis and management of conduction abnormalities after TAVR. As indicated by their titles, the documents were largely based on consensus rather than on hard data. The former document recommended stratifying patients into 1 of 5 categories derived from their baseline and postprocedural electrocardiograms. For each category, the document provides an algorithm distinguishing patients likely to be eligible for discharge within 24-48 ​hours, those at high risk for high-grade AV block, and those requiring pacemaker implantation.^6^ The first attempt to validate this algorithm included patients in the Swiss transcatheter aortic valve implantation registry. The investigators reported that nearly 75% of patients were eligible for early discharge, but also observed that about 1 out of 5 were at high risk for high-grade AV block. Permanent pacemaker implant occurred among 2.7% of patients eligible for early discharge, in 40.9% considered to be at high risk, and obviously in 100% among those in whom the pacemaker was recommended. The authors concluded that the algorithm was generally useful but that the high-risk group was imprecisely defined. It is noteworthy, however, that the overall pacemaker rate in this study was relatively high (16%) and a substantial number of mechanically expandable valves were used.^8^ The latter valve design is no longer available and has been associated with a considerably higher pacemaker rate than either balloon-expandable or self-expanding valves.

In the current issue of Structural Heart, Toshiaki et al. present a rigorous retrospective study that leveraged the recommendations of the Scientific Expert Panel. The investigators studied 808 patients who underwent TAVR with a Sapien S3 valve (Edwards LifeSciences, Irvine, California) between January 2017 and December 2018. At the beginning of this period, they adopted a new implantation technique in which the implant depth was chosen using a caudally angulated right anterior oblique view, referred to as the cusp overlap view, which effectively separates the noncoronary cusp from the right coronary cusp of the aortic valve. As a result of this angulation, the depth of the prosthesis in relation to the membranous septum (and therefore the conduction system) may be assessed with reasonable accuracy compared with the coplanar view, which foreshortens this spatial relationship.^9^ As indicated by the Expert Panel document, the authors stratified the patients into 5 categories: 1) no right bundle branch block and no electrocardiographic (ECG) changes during the TAVR, 2) pre-existing right bundle branch block and no ECG changes during TAVR, 3) ECG changes in patients with pre-existing right or left bundle branch block or intraventricular conduction defect, 4) new onset left bundle branch block, or 5) high-grade or complete heart block during TAVR. The investigators added a sixth category: patients with QRS duration <120 ​ms and normal PR interval, who developed QRS or PR prolongation >20 ​ms but stopped short of an overt conduction abnormality. Electrocardiograms were performed immediately after the procedure and the following day.^10^ In all groups except the first, the Expert Panel document recommends leaving a temporary pacemaker in place at least overnight.^6^ However, the authors found that with these implantation and surveillance techniques, the temporary pacemaker could be removed immediately after the TAVR in 97% of patients with the notable exception of the fifth group (high-grade or complete heart block during TAVR). A total of 6 patients (0.7%) required reinsertion of a temporary pacemaker, while 24 patients (3.0%) required permanent pacemaker during hospitalization. An additional number of 7 (0.9%) patients required permanent pacemakers by 30 days. If one excludes patients in groups 1 (no right bundle branch block or ECG changes) and 5 (intraprocedural high-grade AV block), 98% of temporary pacemakers could be removed earlier than recommended by the Expert Panel and nearly all patients were able to undergo early hospital discharge.^10^

Overall, these results are quite encouraging. Compared with the approximately 10% rate reported in the Transcatheter Valve Therapy Registry of TAVR, a pacemaker rate of 3% is astounding, as it suggests that with appropriate placement techniques and close initial observation, the current pacemaker rate can be reduced by about two-thirds. However, the current report needs to be considered in the proper context, as despite its size, the study is a retrospective compilation from a single center with highly experienced implanters and a crack electrophysiology team. Even before the advent of the cusp overlap technique, there was considerable site-to-site variation in the frequency of pacemaker implant. Several further caveats are important. The patients in this report underwent almost entirely elective procedures and are largely at the lower end of what has been defined as the intermediate risk group. The mean age is 78 years, which is 3 ​years younger than that in the most recently published TVT registry; this group may thus have greater conduction system reserve. Additionally, the cohort consisted entirely of patients receiving balloon-expandable rather than self-expanding valves, which a priori selects a group less likely to receive permanent pacemakers. Finally, although identification of the implant depth vis-a-vis the noncoronary cusp depth in the cusp overlap view is intuitively appealing, the approach has not yet been fully validated. At least one small study has indicated that the implant depth assessed in this view systematically underestimates the depth measured by computed tomography scan.^11^ How should this study be viewed then? It would currently be difficult to argue that these findings should urge each center to mandate an early pacemaker removal program. However, they do provide insight into which groups merit more attention than others and raise the possibility that with careful assessment of early pacemaker removal studies, we will be able to reduce the rate of permanent pacemaker implant following TAVR and will be able to shorten hospital stays significantly.

## Funding

The author has no funding to report.

## Disclosure statement

Neal S. Kleiman reports to be the clinical investigator in clinical trials sponsored by Medtronic and Edwards Lifesciences and reports to be the clinical investigator and also a member of the steering committee in clinical trials sponsored by Abbott and Boston Scientific.
